# Supplementary material for: A Bacterial Form I’ Rubisco Has a Smaller Carbon Isotope Fractionation than Its Form I Counterpart
Source: Biomolecules. 2023 Mar 26;13(4):596. doi: 10.3390/biom13040596 (PMC10135865; doi:10.3390/biom13040596)
Supplement: Supplementary file 1 [file biomolecules-13-00596-s001.zip › biomolecules-2254963-supplementary.pdf]

biomolecules

MDPI

Supplementary Materials

Table S1. Results of rubisco KIE assay.

Rubisco	Time (min)	$\delta^{13}C$ (avg.)	$\delta^{13}C$ (std. err.)	^{13}R (avg.)	^{13}R (std. err.)
L_8	0	−0.592	0.010	0.0111549	$1.81*10^{-7}$
L_8	15	−0.425	0.007	0.0111580	$1.24*10^{-7}$
L_8	30	−0.129	0.021	0.0111634	$3.92*10^{-7}$
L_8	45	0.111	0.026	0.0111679	$4.80*10^{-7}$
L_8	60	0.268	0.017	0.0111708	$3.11*10^{-7}$
L_8	90	0.327	0.010	0.0111718	$1.81*10^{-7}$
L_8	120	0.506	0.007	0.0111751	$1.35*10^{-7}$
L_8	150	0.473	0.012	0.0111745	$1.35*10^{-7}$
L_8	210	0.652	0.013	0.0111778	$2.34*10^{-7}$
L_8	270	0.454	0.007	0.0111742	$1.26*10^{-7}$
L_8	330	0.399	0.006	0.0111732	$1.08*10^{-7}$
L_8	390	0.348	0.014	0.0111722	$2.55*10^{-7}$
L_8S_8	0	0.599	0.026	0.0111768	$4.68*10^{-7}$
L_8S_8	15	0.990	0.017	0.0111840	$3.14*10^{-7}$
L_8S_8	30	1.058	0.008	0.0111853	$1.53*10^{-7}$
L_8S_8	45	1.553	0.015	0.0111944	$2.69*10^{-7}$
L_8S_8	60	1.490	0.010	0.0111932	$1.84*10^{-7}$
L_8S_8	90	1.776	0.015	0.0111985	$2.82*10^{-7}$
L_8S_8	120	1.905	0.013	0.0112009	$2.33*10^{-7}$
L_8S_8	150	1.997	0.011	0.0112025	$1.92*10^{-7}$
L_8S_8	210	1.951	0.009	0.0112017	$1.64*10^{-7}$
L_8S_8	270	1.948	0.008	0.0112016	$1.44*10^{-7}$

Experimental outputs of rubisco KIE assay; $\delta^{13}C$ vs. time is plotted in Figure S1A. Average $\delta^{13}C$ or ^{13}R (n = 10 analytical replicates) is reported with standard error (standard deviation divided by square root of n).

Table S2. Literature compilation of data used to make Figure 1A.

Strain	Form	Phylum	Specificity	KIE	Notes	Specificity Reference	KIE Reference
Prochlorococcus marinus MIT9313	IA	Cyanobacteria	59.9 ± 7.0	24.0 [22.2, 25.6]a	pH 7.5, 25 mM MgCl2, 25C, expressed from E coli	[54]	[24]

Synechococcus elongatus PCC6301	IB	Cyanobacteria	56.1 ± 1.3	22.42 ± 2.37b	pH 8.49, 30 mM MgCl2, 22C, expressed from E coli	[30]	This paper
Synechococcus elongatus PCC6301	IB	Cyanobacteria	50.3 ± 2.0	25.18 ± 0.31b	pH 8.38, 30 mM MgCl2, 22C, expressed from E coli	[54]	[50]
Synechococcus elongatus PCC6301	IB	Cyanobacteria	42.7 ± 2.8	22.0 ± 0.2c	pH 8.1, 25 mM Mg(2+), 25C, expressed from E coli	[55]	[25]
Candidatus Promineofilum breve	I'	Chloroflexi	36.1 ± 0.9	16.25 ± 1.36b	pH 8.52, 30 mM MgCl2, 22C, expressed from E coli	[30]	This paper
Rhodospirillum rubrum	II	Proteobacteria	12.5 ± 0.6	23.0 ± 0.6c	pH 7.9, 25 mM Mg(2+), 25C, expressed from E coli	[55]	[25]
Rhodospirillum rubrum	II	Proteobacteria	12.5 ± 0.6	19.6 ± 0.4c	pH 7.9, 2 mM Mg(2+), 25C, expressed from E coli	[55]	[25]
Rhodospirillum rubrum	II	Proteobacteria	12.5 ± 0.6	22.2 ± 2.1b	pH 8.0, 20 mM MgCl2, room temp?, expressed from E coli (XL1-blue)	[55]	[52]
Rhodospirillum rubrum	II	Proteobacteria	12.5 ± 0.6	17.8 ± 0.8b	pH 7.8, 10 mM MgCl2, 25C, "gift from John Schloss"	[55]	[53]
Candidatus Endorifita persephone	II	Proteobacteria	8.6 ± 0.9	19.5 ± 1.0c	pH 8.0, 30C, 5 mM MgCl2, purified from R pachyptila trophosomes	[44]	[44]

For KIE measurements: Each figure reports uncertainty on the measurement in a different way; superscripts indicate: *a* 95% confidence interval; *b* standard deviation; *c* standard error. Strains for *R. rubrum* not specified in [25,52,53]. We only used data where a pure enzyme, substrate-depletion assay like ours was done. In addition, we only used data from well-characterized strains where rubisco was obtained through expression in *E. coli*. Therefore, we are not including the [46] measurement because it was done in a tobacco plant mutant expressing an *R. rubrum* rubisco sequence *in vivo*, and KIE was calculated by extrapolating to a ratio of intercellular to ambient CO_2 (C_i/C_a) of

1. In addition, we are only showing Form IA/B data from Cyanobacteria and therefore do not include plants or the *Solemya velum* symbiont [36]. Assay temperature was assumed to be room temperature for [52]; rubisco was assumed to be expressed from *E. coli* in [53]. In addition, only the pH 7.9, 25 mM Mg^{2+} condition from [25] was plotted in Figure 1B. See Table 3 in [13] for a recent compilation of all measured KIEs. For Specificity measurements: Most specificity values were not reported with the study, with the exception of this paper and [44,50]. Therefore, specificity values were taken from [30,54] where indicated.

Table S3. Additional Specificity and KIE values used for Figure 1B.

Strain	Form	Specificity	KIE (‰)	Specificity Reference	KIE Reference
Ralstonia eutropha	IC	75	19 [17.5, 20.4]	[56]	[37]
Rhodobacter sphaeroides	IC	60	22.4 [21.1, 24.0]	[56]	[37]
Emiliania huxleyi	ID	79	11.1 [9.8, 12.6]	[57]	[61]
Skeletonema costatum	ID	72.2 ± 2.2	18.5 [17.0, 19.9]	[58]	[62]
Spinacia oleracea	IB	77.2 ± 1.4	30.3 ± 0.8	[59]	[25]
Spinacia oleracea	IB	77.2 ± 1.4	29 ± 1	[59]	[63]
Spinacia oleracea	IB	77.2 ± 1.4	28.2 [26.6, 29.8]	[59]	[36]
Nicotiana tabacum	IB	82.1 ± 0.8	27.4 ± 0.9	[60]	[52]

Data compilation is similar to that used in Figure 4 from [37]. Most specificity values were measured separate from the KIE and are taken from other prior literature [56-60], similar to what was done by [37]. *Solemya velum* gill symbiont (Form IA, KIE = 24.4‰) from [36] was not included because the specificity could not be found. In addition, [25] gives two values at two different assay conditions for *S. oleracea*; here we use the value at pH 8.5, 20 mM $MgCl_2$ but they also report a KIE of 29.0 ± 0.3‰ at pH 7.6, 5 mM Mg^{2+}. KIE values are from [25,36,37,52,61-63]. Error in brackets is reported as mean with 95% confidence intervals; otherwise error is reported as mean ± s.e.

Table S4. Model outputs for converting time to *f*.

Rubisco	Parameter	Estimate	Std. Error	t value	Pr(>|t|)	Signif. Code
L_8S_8	a	$-2.786*10^{-5}$	$2.759*10^{-6}$	-10.097	$1.63*10^{-4}$	***
L_8S_8	b	$1.640*10^{-2}$	$4.310*10^{-3}$	3.804	0.012573	*
L_8S_8	c	$1.120*10^{-2}$	$2.908*10^{-6}$	3852.671	$< 2*10^{-16}$	***
L_8	a	$-2.338*10^{-5}$	$1.369*10^{-6}$	-17.072	$2.58*10^{-6}$	***
L_8	b	$1.769*10^{-2}$	$2.767*10^{-3}$	6.392	$6.90*10^{-4}$	***
L_8	c	$1.118*10^{-2}$	$1.203*10^{-6}$	9291.937	$< 2*10^{-16}$	***

The nonlinear least squares function in R Statistical Software was used for calculation with initial guesses of $a = -1*10^{-5}$, $b = 0.1$, $c = 0.01$ for L_8S_8; $a = -1*10^{-4}$, $b = 0.1$, $c = 0.01$ for L_8. The parameter *c* gives R_{upper} in Equation 1, which then allows time to to be convert to *f*. For L_8S_8, the model found a residual standard error of $1.252*10^{-6}$ on 6 degrees of freedom, required 5 iterations to convergence, and achieved a convergence tolerance of $1.405*10^{-6}$. For L_8, the model found a residual standard error of $1.252*10^{-6}$ on 6 degrees of freedom, required 5 iterations to convergence, and achieved a convergence tolerance of $1.405*10^{-6}$. The significant codes indicate: 0 '***' 0.001 '**' 0.01 '*' 0.05 '.' 0.1 ' ' 1. All analyses were performed using R Statistical Software (v4.1.0; R Core Team 2021, [41]).

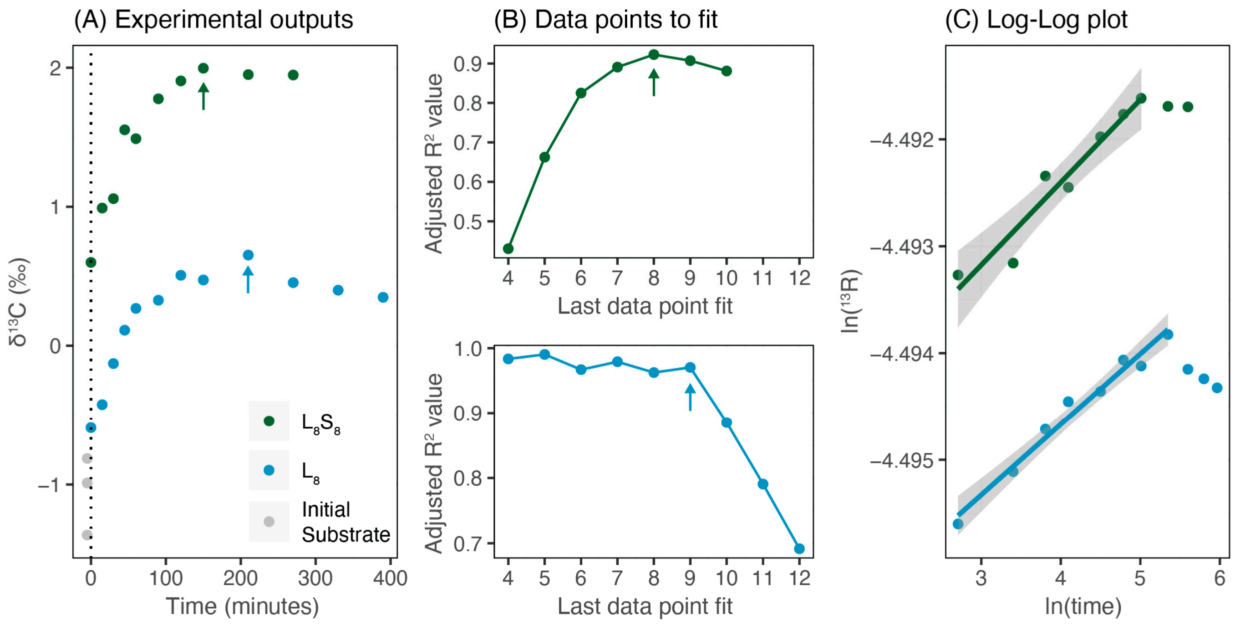

Figure S1. Data preprocessing step. A) Experimental outputs of rubisco KIE assay, showing how the $\delta^{13}C$ of the CO_2 headspace evolves over the experiment. The first time point taken is shown at 0 minutes, and the initial $NaHCO_3$ substrate is shown plotted at -5 minutes for ease of comparison. The arrow indicates the final data point to fit after preprocessing. B) Subplots showing the adjusted R^2 value for the L_8S_8 (above, green) and the L_8 rubisco (below, blue) for linear regressions across different lengths of log-transformed data points. Arrows indicate where the R^2 value starts to decrease (point 8 for L_8S_8 rubisco in green; point 9 for L_8 rubisco in blue); these arrows refer to the same point in Panel A. C) Linear regression across natural log-transformed data to data point 8 for the L_8S_8 rubisco (green) and to data point 9 for the L_8 rubisco (blue). Note the isotopic data is in ^{13}R vs. $\delta^{13}C$ format. The first data point is not plotted because the natural log of zero is undefined. All analyses were performed using R Statistical Software (v4.1.0; R Core Team 2021, [41]). Data visualization was performed using the *ggplot2* package (v3.3.6; Wickham, 2016, [64]).

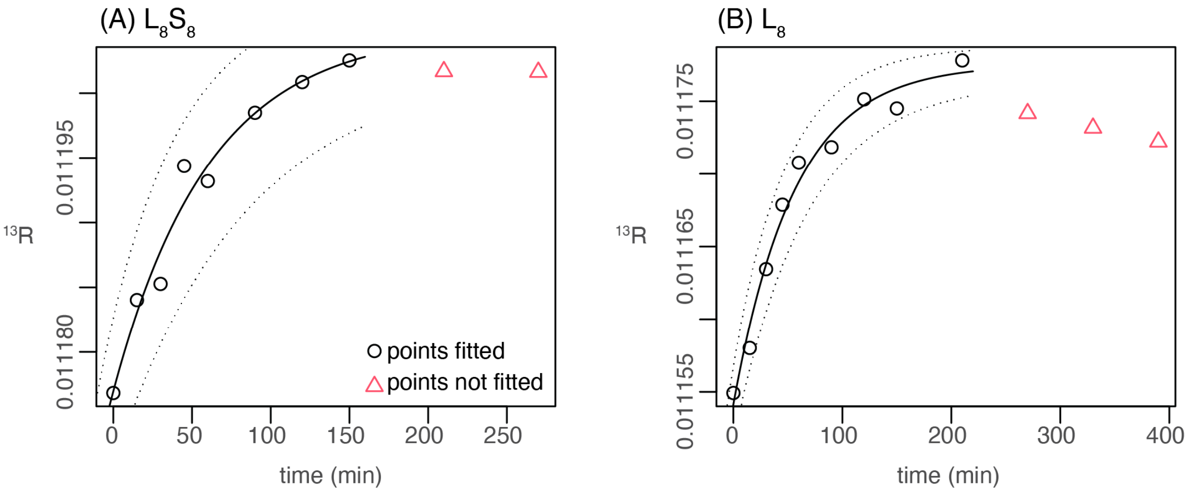

Figure S2. Calculating *f* from time. Plots showing best fit exponential model for (**A**) L_8S_8 vs. (**B**) L_8 rubisco in solid black line. Dotted lines indicate model uncertainty (std. dev.). See Table S3 for best-fit model parameters. Open black circles are points fitted, as determined in Figure S1. Open red

triangles are the points not fit. All analyses and data visualization were performed using R Statistical Software (v4.1.0; R Core Team 2021, [41]).

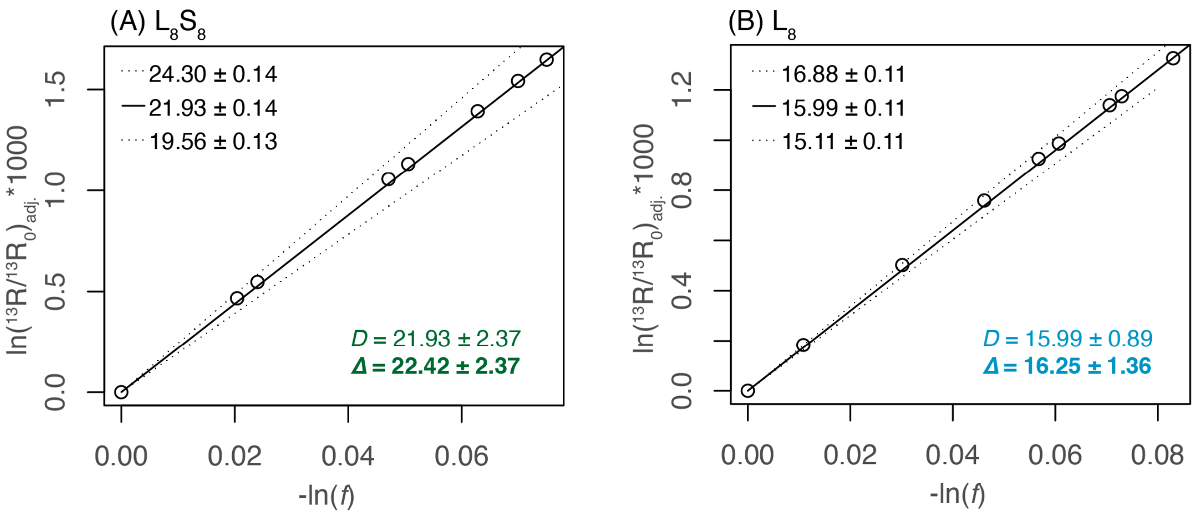

Figure S3. Rayleigh plots with equilibrium adjustment. A) and B) show L_8S_8 and L_8 rubisco with equilibrium adjustment for ^{13}R values (Equation 2; [25]) before linear regression. Solid line gives best fit value using *f* values calculated from the best estimate for parameter *c*. Dotted lines give fit for *f* values calculated using the best estimate ± std. error for *c* as shown in Table S3. Slopes for each line are reported in the upper left corner (best estimate ± std. error). *D* is the slope of the solid, best fit line. Δ is converted from *D* using $\Delta = D/(1-D/1000)$ [25]. All analyses and data visualization were performed using R Statistical Software (v4.1.0; R Core Team 2021, [41]).
